# The prevalence of insufficient iodine intake in pregnancy in Africa: a systematic review and meta-analysis

**DOI:** 10.1186/s13643-022-02072-6

**Published:** 2022-10-27

**Authors:** Charles Bitamazire Businge, Hannibal Tafadzwa Musarurwa, Benjamin Longo-Mbenza, Andre Pascal Kengne

**Affiliations:** 1 Department of Medicine, Faculty of Health Sciences, University of Cape Town, Cape Town, South Africa; 2 Department of Obstetrics and Gynaecology, Faculty of Health Sciences, Walter Sisulu University, Private Bag x1 WSU, Mthatha, 5117 South Africa; 3 Department of Biological Sciences, Faculty of Health Sciences, Walter Sisulu University, Private Bag x1 WSU, Mthatha, 5117 South Africa; 4 Faculty of Medicine, University of Kinshasa, Kinshasa, Democratic Republic of Congo; 5 Non-Communicable Disease Research Unit, South African Medical Research Council, Cape Town, South Africa

**Keywords:** Iodine, Insufficiency, Pregnancy, Africa

## Abstract

**Background:**

Fortification of foodstuffs with iodine, mainly through iodization of salt, which commenced in several African countries after 1995 is the main method for mitigating iodine deficiency in Africa. We assessed the degree of iodine nutrition in pregnancy across Africa before and after the implementation of national iodine fortification programs (CRD42018099434).

**Methods:**

Electronic databases and gray literature were searched for baseline data before implementation of population-based iodine supplementation and for follow-up data up to September 2020. R-metamedian and metamean packages were used to pool country-specific median urinary iodine concentration (UIC) estimates and derived mean UIC from studies with similar features.

**Results:**

Of 54 African countries, 23 had data on iodine nutrition in pregnancy mostly from subnational samples. Data before 1995 showed that severe iodine deficiency was prevalent in pregnancy with a pooled pregnancy median UIC of 28.6 μg/L (95% CI 7.6–49.5). By 2005, five studies revealed a trend towards improvement in iodine nutrition state in pregnancy with a pooled pregnancy median UIC of 174.1 μg/L (95% CI 90.4–257.7). Between 2005 and 2020 increased numbers of national and subnational studies revealed that few African countries had sufficient, while most had mildly inadequate, and some severely inadequate iodine nutrition in pregnancy. The pooled pregnancy median UIC was 145 μg/L (95% CI 126–172).

**Conclusion:**

Improvement in iodine nutrition status in pregnancy following the introduction of fortification of foodstuffs with iodine in Africa is sub-optimal, exposing a large proportion of pregnant women to the risk of iodine deficiency and associated disorders.

**Systematic review registration:**

PROSPERO CRD42018099434

## Background

Iodine deficiency has a spectrum of consequences that not only affect pregnancy outcomes but also subsequent childhood and maternal health [1–3]. Fetal and maternal complications include spontaneous miscarriages, growth restriction, still birth, and maternal postpartum thyroiditis and in cases of severe or persistent iodine deficiency, subclinical and overt hypothyroidism, stunted growth, altered serum lipids, and mental and motor deficits that can affect both mother and child [1–3]. The risk of these complications is higher in settings with endemic iodine deficiency like most countries in Africa in the early 1990s before the initiation of iodine fortification. The degree of iodine deficiency deteriorates in pregnancy due to physiological increase in renal iodine filtration and subsequent loss in urine [4, 5]. Even mild to moderate iodine deficiency during pregnancy is associated with psychomotor and cognitive impairment [1].

Before programs encouraging the fortification of salt and other foodstuffs in Africa, it was estimated that only 10% of the population on the African continent had adequate iodine nutrition [6–8]. This was attributed to low soil iodine content as well as high thiocyanate levels, one of the major goitrogens on the continent [9]. By 2021, surveys using national or subnational samples of school age children (SAC) yielded median urinary iodine concentration (UIC) consistent with adequate iodine intake in most African countries [10]. Despite this, significant disparity in access to iodized salt still exists in several African countries at community level. This is attributed to insufficient capacity by small-scale salt producers to consistently iodize salt, as well as unfavorable trade policies between countries that preclude the importation of affordable iodized salt from neighboring African countries with large production capacity [11].

Due to variation in dietary habits and iodine metabolism of school age children and pregnant women, the school age children median UIC does not accurately predict the degree of iodine nutrition among pregnant women from the same setting [12, 13]. Of the eleven African countries with data on iodine nutrition in pregnancy, five had insufficient, four adequate, and two more than adequate iodine intake in pregnancy [14].

A daily iodine intake of at least 200 μg, up from the recommended 100–150 μg in non-pregnant women, is necessary to cater for the physiological requirements of pregnancy and compensate for the elevated renal losses [15]. In pregnancy a median UIC of < 150 μg/l reflects insufficient intake while UIC of 150–249 μg/l adequate, 250–499 μg/l more than adequate, and UIC>500 μg/l reflecting excessive iodine intake [16]. In the general population and among women in reproductive age at inception of pregnancy, median IUC of < 20 μg/l, 20–49 μg/l, and 50–99 μg/l reflect severe, moderate, and mild iodine deficiency, respectively [15]. The same values may be used to identify populations with pregnant women at risk of moderate and severe iodine deficiency, and 100–149 μg/l for populations with pregnant women at risk of mild iodine deficiency (Table 1) [17]. It is not certain if the iodine fortification efforts have had a significant and sustainable impact on the iodine nutrition status in pregnancy in Africa [18].

**Table 1:** Levels of insufficient iodine intake in pregnancy

Iodine nutrition status	Median Urinary iodine concentration (mUIC)
Insufficient iodine intake	mUIC < 150 μg/L
Moderate-to-severe iodine deficiency	mUIC < 50 μg/L
Mild to moderate iodine deficiency	50 μg/L ≤ mUIC < 150 μg/L

### Rationale

We conducted this systematic review and meta-analysis to ascertain the trend in the prevalence of insufficient iodine nutrition status (median UIC <150 μg/L) among pregnant women in Africa following the implementation of national iodization programs and to establish if this has had a positive impact on the iodine nutrition status of pregnant women in Africa.

## Methods

The methods of this systematic review and meta-analysis were described in a protocol [19] that was also registered with PROSPERO (CRD42018099434). Observational and intervention studies with data on iodine nutrition status in pregnancy conducted in the various African countries were included in this systematic review. The iodine nutrition status was defined according to the WHO/ICCIDD classification of iodine intake of populations using median urinary iodine concentration [15]. All studies are reported in the English or French, or Portuguese languages and conducted on human subjects were considered. We excluded studies conducted among populations of African origin but residing outside Africa, studies lacking prevalence rates and with the absence of data to compute them, and studies not performed in human participants or published in languages other than English, French, and Portuguese. This systematic review is reported in accordance with the Preferred Reporting Items for Systematic reviews and Meta-Analysis (PRISMA) Guidelines [20].

### Search strategy for study identification

#### Electronic searches

We searched PubMed-MEDLINE, Google Scholar, SCOPUS, ISI Web of Science (Science Citation Index), Africa Wide Information, African Index Medicus (AIM), and AFROLIB databases for published studies on iodine deficiency in pregnancy in Africa up to the 30th of September 2020. This search was conducted using a predefined comprehensive and sensitive search strategy combining relevant terms with names of countries in Africa, to obtain the maximum possible number of studies. This search was guided by the African search filter, which has been reported to have good sensitivity (and improved precision) of 74% (1.3–9.4%) and 73% (5–28%) for MEDLINE and EMBASE, respectively [21]. This search filter included names of each African country and shortened terms to capture studies from regions. Countries with official names in a language other than English were entered in the official form, and for countries that have changed names over time, both names were included in the search. The search strategy can be found in the published protocol for this review [19]. We also searched reference lists of relevant citations for articles of interest.

#### Grey literature

We also searched for national ministries of health, international organizations such as the WHO, UNICEF, ICCIDD, IGN, other non-government organisations’ reports, conference, and workshop proceedings using Google Scholar search engine, and major relevant websites such as WHO “African Index Medicus and African Journals Online” (AJOL).

### Study records

#### Data management

All identified studies were entered into endnote software for de-duplication of records. Prior to screening of studies, we created standardized questions according to the inclusion criteria which were pre-tested on a sample of eligible studies.

#### Screening

**Fig. 1:**
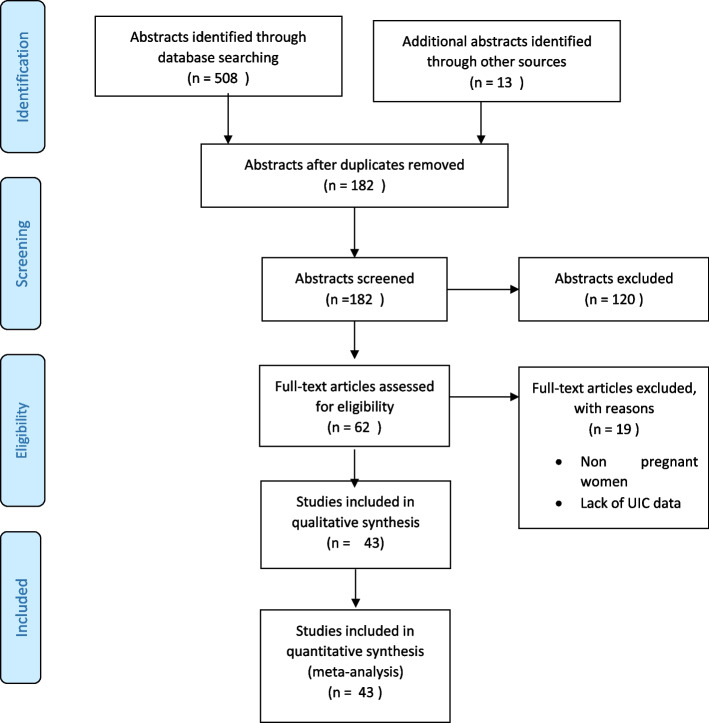
Flow chart showing the processes of selection of studies included in the systematic review

Two investigators (CBB and HM) independently selected studies that meet inclusion criteria. Citations and abstracts were screened for possible inclusion, and duplicate citations were excluded. Titles and abstracts were then screened following the inclusion criteria described above, following which the full texts of potentially eligible articles were obtained. The full texts were then screened using a standardized and pre-tested form to include eligible studies. Disagreements were resolved by consensus, or consultation of a third author (APK). Corresponding authors of potentially eligible studies that did not report the relevant data were contacted. The reasons for exclusion of non-eligible studies were documented. The whole selection process was summarised in a flow chart (Fig. 1).

#### Data extraction

Two investigators (CBB and HM) independently extracted data from included studies, using a standardized and pre-tested data extraction form. Any inconsistencies or disagreement resolved by consensus or consultation with the third investigator (APK).

#### Data items

Data including the year, geographic region, and country where the study was conducted, year of publication, study design, setting (rural or urban, health facility or community-based, national or sub-national), sample size, and the criteria used for determination of the iodine intake were extracted. The median (25th–75th percentiles) and or mean (standard deviation) UIC were recorded.

#### Assessment of methodological quality and risk of bias

Two investigators (CBB and HM) independently scored the quality of included studies. The risk of bias in individual studies was assessed using the Risk of Bias Tool for Prevalence Studies as previously described [19, 22]. Discrepancies were resolved by consensus. The risk of bias and quality scores are presented in Table 2.

**Table 2:** Characteristics of included studies

First author, year	Country, period of data collection	Sample type (national/subnational)	Sample size	UIC median [IQR] or [mean ± sd]	Risk of bias: 0–3 low 4–6 intermediate 7–9 high
**Before 1995**					
Chaouki, 1994 [23]	Algeria (1994)	Sub-national	982	[17.9 ± 0.1]	2 – low
Ngo, 1997 [24]	DRC (1991–1992)	National	306	39.3 [31.1, 52.9]	0 – low
**1995**–**2005**					
Hess, 1999 [25]	Cote de'Ivore	Sub-national	72	351 [74 - 2241]	2 – low
Hess, 1999 [25]	Cote de'Ivore	Sub-national	66	136 [12 - 915]	1 – low
Ojule 1998 [26]	Nigeria, 1998	Sub-national	90	[213.4 ± 9.9]	3 – low
Ojule 1998 [26]	Nigeria, 1998	Sub-national	105	[149 ± 14.7]	3 – low
Dillon, 2000 [27]	Senegal 1996–1997	Sub-national	462	[60 ± 39]	1 – low
Eltom, 2000 [28]	Sudan 1998–1999	Sub-national	47	38 [12.7, 50.8]	3 – low
Lwenje, 2000 [29]	Swaziland	National	165	295 [95% CI 265.5–425.6]	1 – low
**2006**–**2020**					
Akdader-Oudahmane, 2020 [30]	Algeria 2016–2017	Sub-national	173	233 [157, 326]	4 – intermediate
Garnier et al., 2016 [31]	Burkina Faso	National	946	74 [NA, NA]	1 – low
Kavishe et al., 2020 [32]	Burundi 2018	National	87	86.7 [NA, NA]	1 – low
Habimana et al., 2013 [33]	DRC, 2009–2011	Sub-national	225	138 [105, 172]	1– low
IGN, 2017 [14, 34]	Djibouti, 2015 (NS)	National	230	265 [170, 445]	0 – low
MOHP, 2017 [35]	Egypt, 2014–2015	National	1498	135 [NA, NA]	0 – low
Hamza, 2007 [36]	Egypt, 2006	Sub-national	113	[102.9 ± 31.1]	2 – low
Elsayed, 2016 [37]	Egypt,2016	Sub-national	400	170 [NA, NA]	1 – low
Mohammed, 2020 [38]	Ethiopia, 2013–2014	Sub-national	562	120.6 [68.9, 216.4]	1 – low
Fereja, 2018 [39]	Ethiopia	Sub-national	354	85.7 [45.7, 136]	1 – low
Kedir, 2014 [40]	Ethiopia, 2012	Sub-national	435	58.1[21.4, 111.1]	1 – low
Ersino, 2013 [41]	Ethiopia, 2009	Sub-national	172	15 [2.5, 33]	2 – low
Takele, 2017 [42]	Ethiopia, 2017	Sub-national	403	137 [97, 177]	1 – low
Keno, 2017 [43]	Ethiopia, 2014	Sub-national	40	88.6 [66.9, 133.5]	3 – low
Negeri, 2014 [44]	Ethiopia, 2011	Sub-national	423	48 [NA, NA]	2 – low
NaNA, 2019 [45]	Gambia 2018	National	118	113.5 [50.1, 205.9]	0 – low
GHS, 2017 [46]	Ghana, 2015	National	102	183.5 [NA, NA]	0 – low
Gyamfi, 2018 [47]	Ghana, 2016 (ss)	Sub-national	239	159 [NA, NA]	3 – low
Adu-Afarwuah, 2018 [48]	Ghana, 2009–2011	Sub-national	295	137 [78, 221]	2 – low
Farebrother et al., 2018 [49]	Kenya	Sub-national	162	337 [198, 505]	4 – intermediate
Randremanana, 2019 [50]	Madagascar, 2014	National	170	53 [9, 89]	0 – low
Stinca, 2017 [51, 52]	Morocco 2013–2014	Sub-national	245	32 [17, 58]	3 – low
Sadou 2013 [53]	Niger 2012	Sub-national	240	119 [NA, NA]	2 – low
Hess, 2016 [54]	Niger, 2014–2015	Sub-national	662	69 [38.1, 114.3]	1 – low
Jibril, 2016 [55]	Nigeria, 2014	Sub-national	300	193 [NA, NA]	2 – low
Kayode, 2019 [56]	Nigeria, 2012	Sub-national	133	135 [NA, NA]	1 – low
Ujowundu, 2010 [57]	Nigeria, 2009	Sub-national	302	[152.09 ± 41.65 ]	2 – low
Rohner, 2016 [58]	Sierra Leone, 2013	National	154	175.8 [NA, NA]	0 – low
MOH-FGS, 2020 [34]	Somalia, 2018–2019	National	236	369 [142.9, 752]	0 – low
Mabasa, 2019 [59]	South Africa, 2012–2013	Sub-national	565	164 [92, 291]	3 – low
Stinca, 2017 [51, 52]	South Africa	Sub-national	207	174 [95.3, 297.6]	3 – low
Mtumwa, 2017 [60]	Tanzania, 2009–2010	National	947	136.8 [58.8, 258]	1 – low
Ba, 2020 [61]	Tanzania, 2015–2016	National	266	156.1 [64.6, 260.4]	0 – low
Stinca, 2017 [51, 52]	Tanzania, 2016	Sub-national	330	422 [270, 609]	3 – low
Chinyanga, 2006 [62]	Zimbabwe, 2006	Sub-national	94	115.5 [43, 225]	4 – intermediate

#### Data synthesis, analysis and assessment of heterogeneity

**Fig. 2:**
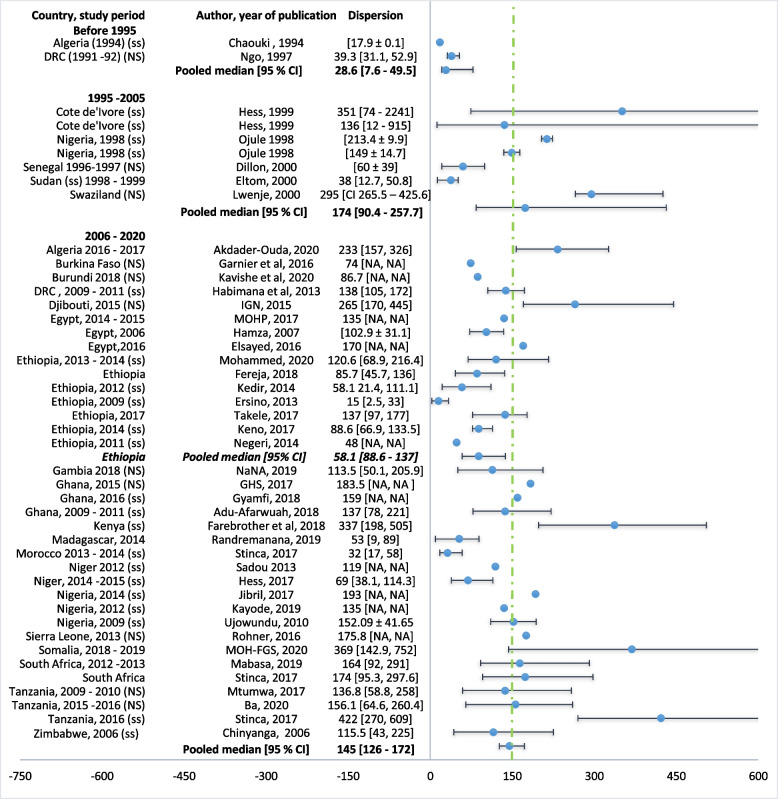
Median urinary iodine concentration (IUC μg/L) of pregnant women for studies conducted before 1995, between 1995–2004 and 2005–2020 (the dashed vertical line shows adequate median UIC during pregnancy; a [b, c] denotes median with IQR, a [±b] denotes mean and standard deviation, and a [b – c] median with the range). NS national survey, ss sub-national survey

Prevalence data was summarized by country and period of study (Table 2). Median pregnancy IUC were pooled using R meta-median package. Sub-group analysis was carried out according to the time the studies were conducted that is before 1995, between 1995–2004 and 2005–2020 (Fig. 2) In order to check for heterogeneity and publication bias, the mean UIC and standard deviation were derived from the median UIC using the methods described elsewhere [63, 64] (Table 3). The derived means were then pooled using metamean R package and degree of heterogeneity between the included studies and the difference in the mean of subgroups estimated. Publication bias was assessed using a funnel plot and an accompanying linear regression test.

**Table 3:** Means and standard deviations (SD) derived from the medians of the included studies

First author, year	Country, period of data collection	Sample size	median	Derived mean	Derived SD
Chaouki, 1994 [23]	Algeria (1994)	982	17.9	17.9	0.1
Ngo, 1997 [24]	DRC (1991–1992)	306	39.3	43.7	21.5
Hess, 1999 [25]	Cote de'Ivore	72	351	351	455
Hess, 1999 [25]	Cote de'Ivore	66	136	184.2	192.3
Ojule 1998 [26]	Nigeria, 1998	90	213	213	9.9
Ojule 1998 [26]	Nigeria, 1998	105	149	149	14.7
Dillon, 2000 [27]	Senegal 1996–1997	462	60	60	39
Eltom, 2000 [28]	Sudan 1998–1999	47	38	33.6	29.1
Lwenje, 2000 [29]	Swaziland	165	295	299	30
Akdader-O, 2020 [30]	Algeria 2016–2017	173	233	234.8	37
Garnier, 2016 [31]	Burkina Faso	946	74	74	28.2
Kavishe, 2020 [32]	Burundi 2018	87	86.7	87	33.2
Habimana, 2013 [33]	DRC, 2009–2011	225	138	138.4	50
IGN, 2017 [14, 34]	Djibouti, 2015 (NS)	230	265	294	205
MOHP, 2017 [35]	Egypt, 2014–2015	1498	135	135	50.5
Hamza, 2007 [36]	Egypt, 2006	113	102	102.9	31.1
Elsayed, 2016 [37]	Egypt,2016	400	170	173.5	56.5
Mohammed, 2020 [38]	Ethiopia, 2013–2014	562	120.6	136.1	109.6
Fereja, 2018 [39]	Ethiopia	354	85.7	89.3	67.2
Kedir, 2014 [40]	Ethiopia, 2012	435	58.1	63.8	66.7
Ersino, 2013 [41]	Ethiopia, 2009	172	15	16.9	22.8
Takele, 2017 [42]	Ethiopia, 2017	403	137	137	59.5
Keno, 2017 [43]	Ethiopia, 2014	40	88.6	96.8	51.2
Negeri, 2014 [44]	Ethiopia, 2011	423	48	48	17.9
NaNA, 2019 [45]	Gambia 2018	118	113.5	123.7	116.7
GHS, 2017 [46]	Ghana, 2015	102	183.5	183.5	69.5
Gyamfi, 2018 [47]	Ghana, 2016 (ss)	239	159	159	59.7
Adu-Afarwuah, 2018 [48]	Ghana, 2009–2011	295	137	145.8	106.5
Farebrother, 2018 [49]	Kenya	162	337	347.2	229.6
Randremanana, 2019 [50]	Madagascar, 2014	170	53	50.2	59.8
Stinca, 2017 [51, 52]	Morocco 2013–14	245	32	35.9	30.6
Sadou 2013 [53]	Niger 2012	240	119	119	44.8
Hess, 2017 [54]	Niger, 2014–2015	662	69	73.9	56.4
Jibril, 2016 [55]	Nigeria, 2014	300	193	193	71.5
Kayode, 2019 [56]	Nigeria, 2012	133	135	138.5	58.5
Ujowundu, 2010 [57]	Nigeria, 2009	302	151.1	152.1	41.7
Rohner, 2016 [58]	Sierra Leone, 2013	154	175.8	176	65.9
MOH-FGS, 2020 [34]	Somalia, 2018–19	236	269	424	454
Mabasa, 2019 [59]	South Africa, 2012–13	565	164	183.3	147.9
Stinca, 2017 [51, 52]	South Africa	207	174	189.8	151
Mtumwa, 2017 [60]	Tanzania, 2009–2010	947	136.8	151.9	147.9
Ba, 2020 [61]	Tanzania, 2015–2016	266	156.1	160.6	146
Stinca, 2017 [51, 52]	Tanzania, 2016	330	422	434.3	252.4
Chinyanga, 2006 [62]	Zimbabwe, 2006	94	115.5	128.5	137

## Results

Figure 1 shows the PRISMA flow chart of the study selection process. A total of 521 abstracts were identified from the searches. After removing duplicates, the titles and abstracts of 182 articles were screened for eligibility. Of these, 62 full-text articles were accessed and screened out of which 42 studies met the inclusion criteria and were included in the meta-analysis [23–62, 65–67].

### Characteristics of included studies

Out of the 42 studies, two were carried out before 1995, five between 1995 and 2005, and thirty-five between 2006 and 2020. Only eleven of the forty-two studies had data derived from national representative samples. The internal and external validity of the included studies were determined using a 9-point score (Table 2). Most of the studies (37/42) had a low risk of bias with the rest having intermediate risk (Table 2).

### The prevalence of insufficient iodine intake (UIC <150 μg/L) among pregnant women on the various African countries before 1995, 1995–2005, and 2006–2020

Before 1995, available data from two studies revealed moderate countrywide iodine deficiency in pregnancy in the Democratic Republic of Congo at the time and severe iodine deficiency in pregnancy in a subnational sample from North-Eastern Algeria [23, 24]. The pooled median UIC across the two studies was 28.6 μg/L (95% CI 7.6–49.5), with considerable heterogeneity (*I*^2^ 99.73 %, *p* < 0.001, Fig. 2).

Between 1995 and 2005, four subnational studies from Ivory coast, Nigeria Sudan, and Senegal, and one national survey from Swaziland [25–29] yielded a pooled pregnancy UIC of 174.1 μg/L (95% CI 90.4–257.7, Fig. 2), with considerable heterogeneity (*I*^2^ 99.96 %, *p* < 0.001).

Between 2005 and 2020, 35 studies from 18 countries had pregnancy median UIC data. Eleven of the studies were national surveys from 10 countries. These national surveys revealed more than adequate intake in Djibouti and Somalia [34, 65]; adequate iodine intake in Ghana, Sierra Leone, and Tanzania [46, 58, 61]; mild inadequate intake in Egypt, Gambia, and Tanzania [35, 60], and moderate insufficient iodine intake in Burkina Faso, Burundi, and Madagascar [31, 32, 50]. The remaining studies [30, 33, 36–44, 47–49, 51–57, 59, 62, 66, 67] were subnational studies. The pooled median pMUIC across the 35 studies conducted between 2005 and 2020 was 145 μg/L (95% CI 126–172), with substantial heterogeneity (*I*^2^ 99.81%, *p* < 0.001) (Fig. 2). There was a significant increase in pregnancy median UIC between 1995 and 2020 compared to the period before 1995 (Kendaull’s tau correlation co-efficient 0.270, *p* = 0.032).

### Derived mean UIC by time-period

**Fig. 3:**
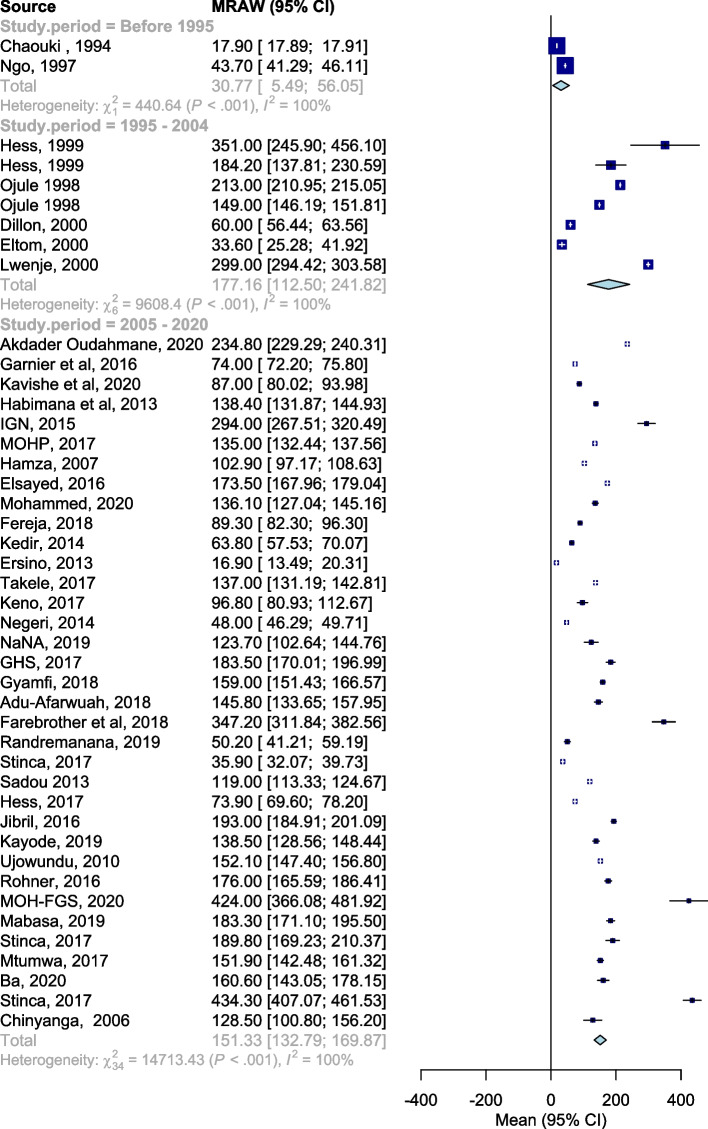
Forest plot showing subgroup analysis of derived mean UIC (μg/L) of the studies conducted before 1995, 1995 to 2005, and 2005 to 2020

The pooled derived mean pregnancy UIC (Table 2, Fig. 3) was 27.96 μg/L (95% CI 11.6–67.04, tau 0.630) before 1995; 143.22 μg/L (95% CI 108.65–188.78, tau 0.362) between 1995 and 2005; and 127.99 μg/L (95% CI 108.59–150.85, tau 0.493), with significant difference across time-period (*Q* = 12.24, d.f. = 2, *p* = 0.002).

### Assessment of publication bias

**Fig. 4:**
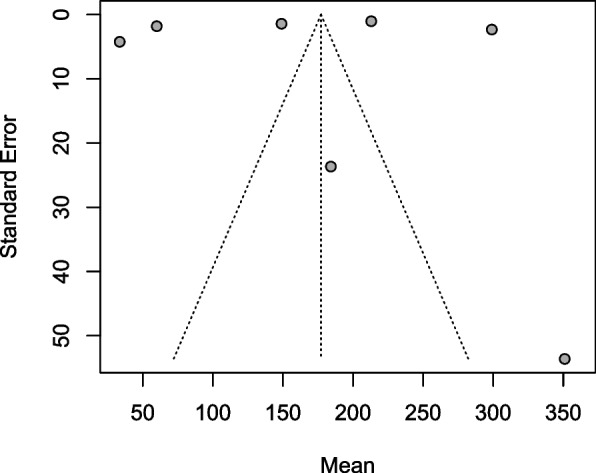
The funnel plot of the studies carried out between 1995 and 2004

**Fig. 5:**
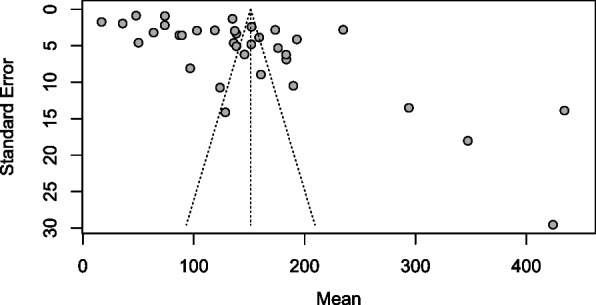
The funnel plot of the studies carried out between 2005 and 2020

**Fig. 6:**
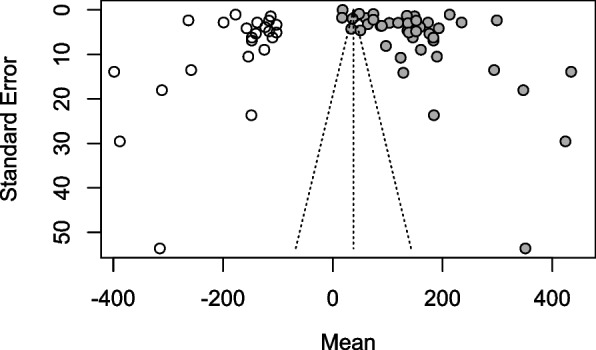
The trim and fill funnel plot of the studies carried out between 2005 and 2020

Publication bias was assessed using funnel plots. The funnel plot for the studies in the period 1995–2004 was not suggestive of potential publication bias (Fig. 4) (R metabias linear regression test *t* = −0.36005, *p* value = 0.7335). No additional studies were imputed after checking for funnel asymmetry using the Twedie and Duval’s trim and fill test. The funnel plot for the studies carried out between 2005 and 2020 was asymmetrical (Fig. 5). The trim and fill test imputed sixteen potential missing studies suggesting potential publication bias (Fig. 6). The funnel plot asymmetry was confirmed by the R metabias linear regression test (*t* = 3.872, *p* < 0.001).

## Discussion

This review found that pregnant women in Africa had moderate to severe iodine deficiency before the implementation of iodine supplementation in 1995. Mild to moderate iodine deficiency in pregnancy was still prevalent in several regions of various African countries by 2005, the year designated for elimination of iodine deficiency globally. However, there was significant improvement in the iodine nutrition status in pregnancy in Africa between 2005 and 2020 compared to the period before 1995 although this is still insufficient (median pMUIC < 150 μg/L). Overall, there is paucity of nationwide representative data on iodine nutrition status in pregnancy in Africa. In order to ensure successful elimination of iodine deficiency disorders, the World Health Organization recommends regular monitoring of iodine nutrition status at national level as well as for high-risk populations such as pregnant women, lactating mothers, and children 7–24 months of age [15]. Pregnancy median UIC data was available for about 50% of the African countries with most derived from sub-national samples. This can be overcome by including the assessment of the iodine nutrition status in the national demographic health surveys.

The limited available data before 1995 showed that some African countries had moderate to severe regional or nationwide iodine deficiency in pregnancy [23, 24]. This may reflect the continental iodine nutritional status in pregnancy at that time since only about 10% of the general population in Africa had adequate iodine nutrition before 1995 [6]. Protracted iodine deficiency predisposes to severe thyroid hyper-stimulation, which together with the prevalent dietary thiocyanates and nitrates in several African countries leads to inflammation, infiltration by immune cells, and oxidative damage to thyroid parenchyma and necrosis [68]. This is exacerbated by the increased loss of iodine through urine during pregnancy, which could account for the disproportionately higher rates of thyroid diseases among women [4, 7].

Following the initiation of iodine fortification of foodstuffs in most countries in 1995 and thereafter, the World Health Organization (WHO) earmarked the 2005 as the year for elimination of iodine deficiency globally [15]. Although the current study found a pooled UIC of 174.1μg/L from eligible studies conducted between 1995 and 2005, which is suggestive of sufficient iodine intake during pregnancy, the number of studies was small and therefore not representative of all the pregnant women in Africa during this period. The studies also revealed that in several countries, there were areas with optimum and others with insufficient iodine nutrition status in pregnancy. This demonstrates lack of equity in implementation of iodine deficiency mitigating strategies within individual countries. This could partly have been due to the dependence on median school age UIC (SAC UIC) as a surrogate measure of national iodine nutrition status. Median SAC UIC does not to accurately estimate iodine nutrition state in pregnancy [11, 69]. Hence, in areas with marginally sufficient iodine intake as estimated using median SAC UIC, pregnant women and their unborn babies may still be at high risk of iodine deficiency. However, the level of iodine insufficiency as revealed in studies conducted between 1995 and 2005 was marginal compared to countries with data before 1995 implying a significant positive impact of iodine fortification on the degree of iodine deficiency in pregnancy in Africa.

Between 2005 and 2020, an increased number of national and sub-national surveys were conducted to assess the iodine nutrition status in pregnancy in several African countries. Some regions within individual countries had sufficient while others had various degrees of insufficient iodine intake in pregnancy more than 20 years after implementation of iodine fortification. Some of these subnational surveys revealed a pregnancy median UIC marginally above the sufficient level. This implies that large proportions of pregnant women may still be at risk of iodine deficiency and its attendant adverse effects. This calls for establishment and strengthening of iodine nutritional status monitoring mechanisms through collaborative efforts of national health departments as well as health related local and international non-governmental organizations. Populations still at risk of moderate to severe iodine deficiency would benefit from iodine supplementation such an annual dose of iodized oil until iodine deficiency is eliminated through equitable access to adequately iodized salt and other national food fortification programs [15]. Further research and innovative strategies to overcome barriers to equitable access of iodised salt such as parallel distribution and use of non-iodized salt in the food industry need further consideration.

### Strengths and limitations

To our knowledge, this is first systematic review aiming at assessing the level of iodine deficiency among pregnant women in Africa from the time of initiation of iodine supplementation to September 2020. This review was limited by the small number of studies before 1995 and by the subnational nature of the majority of studies conducted after 1995 most of which were from small geographical locations within the African countries hence not representative of national populations.

## Conclusion

There is still paucity of data on iodine nutrition status in pregnancy from half of the countries in Africa. The available data shows a significant but inadequate improvement in the iodine nutrition status of pregnant women in several African countries after 1995. A few countries still have moderate to severe iodine deficiency in pregnancy at national or regional more than two decades after implementation of iodine food fortification. The inclusion of iodine nutrition assessment in national demographic surveys will help identify populations and geographical locations that may need iodine supplementation as well as regularly monitor the effectiveness of national iodization programs. Legal frameworks and trade policies that regulate the production and trade in iodized salt within and between countries need to be reviewed so as to foster a sustainable production and supply of adequately iodized salt at community level.

## Abbreviations

ICCIDD: International Council for Control of Iodine Deficiency Disorders
IGN: Iodine Global Network
PRISMA-P: Preferred Reporting Items for Systematic Reviews and Meta-Analysis Protocols
pMUIC: Pregnancy median urinary iodine concentration
SAC: School age children
STROBE: Strengthening the Reporting of Observational Studies in Epidemiology
UIC: Urine iodine excretion
WHO: World Health Organization
UNICEF: United Nations Children’s Education Fund
UIC: Urinary iodine concentration
USI: Universal salt iodization
